# The Effects of Probiotic Supplementation on the Incidence of Diarrhea in Cancer Patients Receiving Radiation Therapy: A Systematic Review with Meta-Analysis and Trial Sequential Analysis of Randomized Controlled Trials

**DOI:** 10.3390/nu11122886

**Published:** 2019-11-27

**Authors:** Navin Kumar Devaraj, Subapriya Suppiah, Sajesh K. Veettil, Siew Mooi Ching, Kai Wei Lee, Rohit Kunnath Menon, Man Jun Soo, Inas Deuraseh, Fan Kee Hoo, Dhashani Sivaratnam

**Affiliations:** 1 Department of Family Medicine, Faculty of Medicine and Health Sciences, Universiti Putra Malaysia, Selangor 43400, Malaysia; knavin@upm.edu.my (N.K.D.); lee_kai_wei@yahoo.com (K.W.L.); benmanjun@gmail.com (M.J.S.); rmd.inas@gmail.com (I.D.); 2 Department of Radiology, Faculty of Medicine and Health Sciences, Universiti Putra Malaysia, Selangor 43400, Malaysia; subapriya@upm.edu.my; 3 School of Pharmacy, International Medical University, Kuala Lumpur, Malaysia, Kuala Lumpur 57000, Malaysia; 4 School of Dentistry, International Medical University, Kuala Lumpur, Malaysia, Kuala Lumpur 57000, Malaysia; RohitKunnath@imu.edu.my; 5 Department of Medicine, Faculty of Medicine and Health Sciences, Universiti Putra Malaysia, Selangor 43400, Malaysia; fan_kee@upm.edu.my; 6 Department of Surgery, Faculty of Medicine and Health Sciences, Universiti Putra Malaysia, Selangor 43400, Malaysia; dhashani@upm.edu.my

**Keywords:** probiotics, randomized controlled trials, placebo, radiation-induced diarrhea, chemotherapy, trial-sequential analysis

## Abstract

The protective effects of probiotic supplementation against radiation-induced diarrhea (RID) have been reported in previous systematic reviews; however so far, only non-conclusive results have been obtained. The objective of this study was to systematically update and evaluate the available evidence for probiotic supplementation. The protocol of this systematic review has been registered (CRD42018106059) with the International Prospective Register of Systematic Reviews (PROSPERO). The primary efficacy outcome was the incidence of RID. Secondary outcomes were the incidence of watery stool, soft stool, and antidiarrheal medication use. There were eight trials, and a total of 1116 participants were included in the primary analysis. Compared with placebo, probiotics were associated with a lower risk of RID [risk ratio (RR) = 0.62, 95% CI = 0.46, 0.83]. A requisite heterogeneity-adjusted trial sequential analysis indicated conclusive evidence for this beneficial effect. No statistically significant reduction in RID (RR = 0.52, 95% CI = 0.14, 1.91) was observed on subgroup analysis in patients receiving both radiation therapy and chemotherapy. However, those patients receiving only radiation therapy (RT) demonstrated significant benefit (RR = 0.61, 95% CI = 0.48, 0.78). There was a significant difference in the antidiarrheal medication use (RR = 0.54, 95% CI = 0.35, 0.84) observed with the use of probiotics. However, no significant difference was observed for the incidence of soft and watery stool. The use of probiotics is beneficial in preventing RID in patients receiving RT.

## 1. Introduction

Radiation therapy (RT) is a treatment strategy that conveys energy to eradicate malignant cells in the area specifically targeted by the physician [1]. In various settings, RT may be the solitary treatment, or it has been used with surgery and chemotherapies for a wide range of cancers. Particularly in the pelvic region, prostate, gynecological, and colorectal cancers are among the common type of malignancies that may require RT alone or in combination with other treatment strategies [2]. It is now recognized as a main treatment option to antagonize the unopposed development and progression of the aforementioned cancers. Despite its effectiveness, RT can be associated with important acute side effects including diarrhea that can occur at any time during or shortly after treatment. Radiation-induced diarrhea (RID) often appears during the third week of treatment, with reports of occurrence ranging from 20 to 70 percent [3], and may then have a significant negative influence on the patient’s quality of life. However, at present, there are no effective preventive strategies for RID.

Probiotics, which are recognized primarily as a supplement, are increasingly being consumed for the promotion of gut health and also to shorten the duration of diarrheal illness. Probiotics are defined as live micro-organisms, which, when administered in adequate amounts, confer the host with a series of health benefits as defined by the World Gastroenterology Organization (WGO) practice guideline [4]. So far, several randomized clinical trials (RCTs) have explored its benefits on RID. Recent meta-analyses (using five to six RCTs) demonstrated some evidence supporting the beneficial effects of probiotics—their ability to reduce the incidence of RID [5,6]. A systematic analysis and meta-analysis by Wang et al. involving nine RCTs and placebo-controlled studies with 1265 participants showed the net beneficial effect of probiotics as compared to placebo in the reduction in chemoradiotherapy-induced diarrhea (OR = 0.47, 95% confidence interval 0.28–0.76 and p = 0.002) [7]. Further, a recently published systematic review by Bowen et al. provided yet another strong case to promote the use of *Lactobacillus* spp. containing probiotics for the prevention of chemoradiotherapy- or radiotherapy-induced diarrhea in patients afflicted with pelvic malignancy [8]. However, vast heterogeneity of data was observed in the studies that were included in the meta-analyses. Moreover, the trial included in this meta-analysis that demonstrated a low risk of bias denied the beneficial effect of probiotics on RID [9]. Hence the beneficial effects observed for probiotics in these meta-analyses would be due to the possible effects of random errors. In a meta-analysis that only has a small number of patients and trials, positive conclusions can be secondary to random errors as an effect of the play of chance (random error) rather than to a ‘true’ intervention effect [10]. Trial sequential analysis (TSA) integrates the risks of random errors and determines the required boundaries and sample size that consider whether conclusive evidence in a meta-analysis has been achieved [11]. Furthermore, additional trials [12,13] have become available since the last meta-analysis, allowing the existing evidence for probiotics on RID to be re-examined. 

Hence, the objective of this review was to systematically update the effects of probiotics on the incidence of RID among cancer patients receiving RT. We performed meta-analyses coupled with TSA in order to quantify the reliable and conclusive evidence of probiotics. By employing the Grading of Recommendations, Assessment, Development and Evaluation (GRADE) approach, we also summarized the available evidence on the use of probiotics. 

## 2. Methods 

### 2.1. Design and Data Sources

The Cochrane Handbook for Systematic Reviews of Interventions was used as the research guide for the overall conduct of this meta-analysis [14] and to ensure that this study is in compliance with the Preferred Reporting Items for Systematic Reviews and Meta-Analyses (PRISMA) statement [15]. The protocol of this systematic review has been registered (registration number: CRD42018106059) with the International Prospective Register of Systematic Reviews (PROSPERO) previously.

We identified relevant studies by a systematic search of MEDLINE (Via Ovid), MEDLINE In-Process and Other Non-Indexed Citations (Via Ovid), Cochrane CENTRAL Register of Controlled Trials, Embase (Via Ovid), and PubMed from inception to December 2018. We initially developed a search strategy in MEDLINE and subsequently modified it for the other databases (Appendix A.1). The reference lists of published systematic reviews and identified articles was also double-checked to categorize the studies that the existing database searches did not capture. The studies included were RCTs and those that meet the following inclusion criteria: participants were adult humans who underwent radiotherapy; intervention involved the mandatory use of probiotics; comparators were placebo with or without other base ingredients; and the outcome was the proportion of participants who developed RID. The primary efficacy outcome of interest was the incidence of RID. Secondary outcomes were the incidence of soft stool, watery stool, and antidiarrheal medication use.

### 2.2. Data Extraction and Quality Assessment

Requisite data were extracted independently and in duplicate by two reviewers into a standardized data extraction form. The extracted data contained: first author, publication year, location of study, the sample size of two groups, mean age of participants, primary tumor site, type of therapy, any accompanying chemotherapy, and probiotics (microbial strain and dose). The intention-to-treat principle has been used for all outcomes—in which, we used the initial number of participants randomized to each trial arm and performed the analyses irrespective of how the authors of the original trials had analyzed their data previously [14]. Therefore, participants who were lost to follow-up were considered free of any relevant outcomes including RID.

Two reviewers (KWL, MJS) used the Cochrane risk of bias instrument to independently assessed the risk of bias within each study [16]. We evaluated all the sources of bias including sequence generation, allocation concealment, blinding of personnel and participants, blinding of outcome assessment, incomplete outcome data, selective outcome reporting, and others. These both reviewers resolved any disagreements by having a discussion with the review team (NKD, SS, RKM, FKH, DS), and one of two arbitrators (SKV and SMC) adjudicated any unsolved disagreements between these reviewers (Supplement 1 for Risk of bias graph and Supplement 2 for Risk of bias summary). 

### 2.3. Statistical Analysis

Meta-analyses were performed using a random-effects model to estimate the effect size such as the pooled relative risk (RR) and 95% confidence intervals (CI), incorporating both between and within-study heterogeneity. Heterogeneity between trials was assessed by using the I^2^ statistic [14]. A substantial level of heterogeneity was interpreted when the I^2^ estimate was greater than or equal to 50%. We assessed publication bias and small-study effects using funnel plot asymmetry testing and Egger’s regression test, respectively [17]. The intention-to-treat principle was used for all analyses. We performed multiple pre-specified sensitivity analyses by restricting studies with low risk of bias and using per-protocol completer analysis to assess the robustness of our primary efficacy outcome. Subgroup analyses were performed for the primary outcome for those patients who received RT alone and those receiving combination therapy (i.e., RT with chemotherapy). For statistical analysis, we used Stata version 15.1 (StataCorp, College Station, TX, USA). The Grading of Recommendations, Assessment, Development and Evaluation (GRADE) approach was used to rate the quality of evidence (very low, low, moderate and high) of estimates derived from meta-analyses using GRADEpro version 3.6.1 (McMaster University, 2014) [18]. 

Type-I errors may occur in meta-analyses due to an increased risk of random error when smaller numbers of patients and RCTs are recruited, and due to repeated significance testing when a cumulative meta-analysis is updated with the latest RCTs [10,11]. We performed trial sequential analysis for primary outcome using the TSA software package (available at http://www.ctu.dk/tsa/; produced by Copenhagen Trial Unit, Center for Clinical Intervention Research, Rigshospitalet, Copenhagen, Denmark in order to avoid random errors in our meta-analysis. TSA will determine whether the evidence in our meta-analysis is reliable and conclusive by providing the necessary sample size for our meta-analysis and boundaries. 

### 2.4. Operational Definitions 

The Bristol scale was used to classify the form of human feces into seven categories, namely, grade 1 = normal stools (Bristol 1–4); grade 2 = soft stools, in pieces (Bristol 5–6); grade 3 = liquid stools, no shape (Bristol 7). The detailed description for each grade is: grade 1 = severe constipation (separate hard lump); grade 2 = mild constipation (lumpy and sausage like); grade 3–4 = normal (a sausage shape with cracks in the surface or like a smooth, soft sausage or snake); grade 5 = lacking fiber (soft blobs with clear-cut edges); grade 6 = mild diarrhea (mushy consistency with ragged edges); grade 7 = severe diarrhea (liquid consistency with no solid pieces) [19,20].

## 3. Results

### 3.1. Description of Included Trials

Study selection, inclusion, and exclusion at each screening phase for the efficacy end points are described in Appendix A.2. The characteristics of the included studies are shown in Table 1. Table 2 describes the probiotics used.

### 3.2. Population Characteristics 

The mean age of the participants ranged from below 18 years to 75 years [9,12,13,21,22,23,24,25]. Five studies exclusively discuss gynecological cancers including uterine and cervical cancer [9,13,22,24,25] and the rest discuss n other abdominal pelvic tumors including sigmoid, colorectal, prostate and bladder cancer in addition to the gynecological cancers [12,21,23]. Patients in all the trials receive radiotherapy, and three of the trial participants also receive chemotherapy [9,22,25]. The total radiation dosages ranged from 40 to 4000 to 5000 cGy which is the unit of absorbed radiation dose of ionizing radiation, e.g. X-rays. 

### 3.3. Intervention Characteristics 

The main type of probiotics given was *Lactobacillus*, *Bifidobacterium* and *Streptococcus*, with dosages ranging from 1 × 10^9^ to 1.35 × 10^12^ CFU. The dosages of probiotics ranged to bd (twice a day) to tds (three times a day) in dose. In total, 538 patients receive probiotics while 479 patients receive placebo treatment.

### 3.4. Quality Assessment (ROB)

Out of the eight trials, four trials were of high risk [9,21,22,23], and one trial was of low risk [21]. In total, three trials were of some concern or uncertain risk of bias [12,13,24].

### 3.5. Primary Outcome: Risk of RID

**Figure 1:**
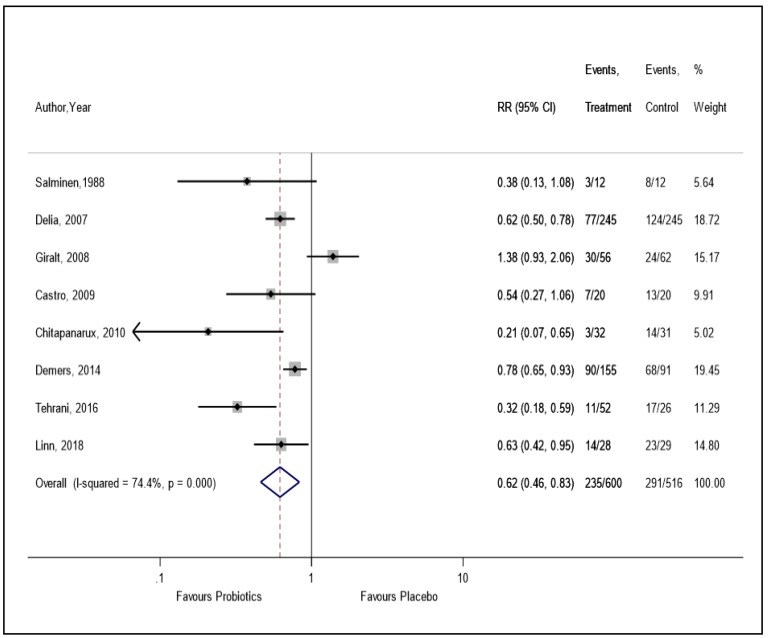
The effect of probiotics on the incidence of radiation-induced diarrhea.

Based on primary meta-analysis using eight RCTs (n = 1,116), the use of probiotics reduced the risk of RID compared to placebo (RR = 0.62, 95% CI = 0.46, 0.83, I^2^ = 74.4%), with significant heterogeneity (Figure 1). Evidence of publication bias was observed in the funnel plot asymmetry test (Appendix A.3). However, Egger’s regression test demonstrated no evidence of small-study effects (Appendix A.4).

**Figure 2:**
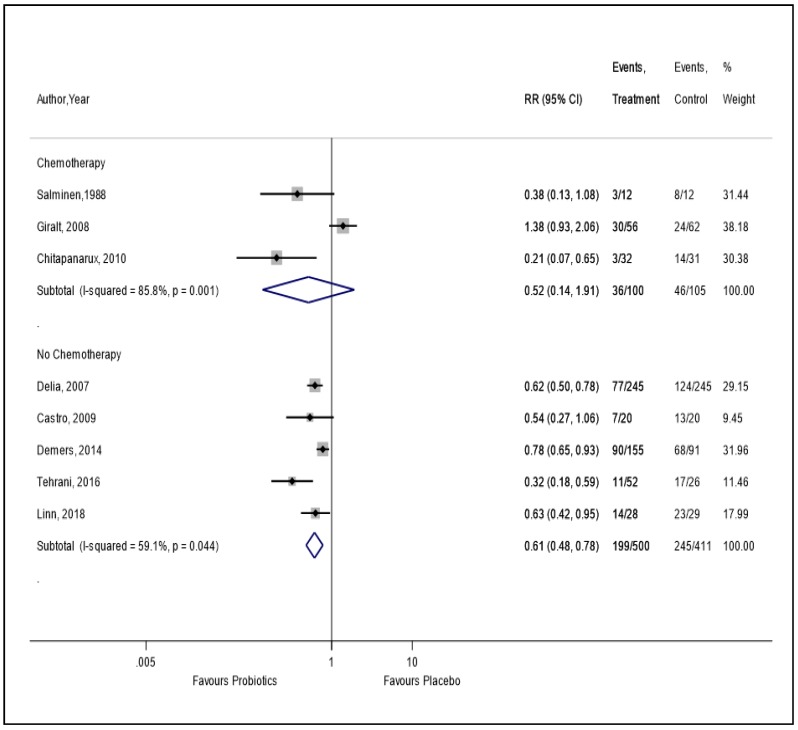
The effect of probiotics on the incidence of radiation-induced diarrhea in patients receiving radiotherapy with or without chemotherapy.

In the sensitivity analysis, by excluding four trials [9,21,22,23] with a high risk of bias, we found a 56% reduction in RID (RR = 0.44, 95% CI = 0.28, 0.70) in patients who were administered supplemental probiotics versus placebo, with a moderate level of heterogeneity (I^2^ = 50.1%) (Appendix A.5). The finding from the sensitivity analysis by per-protocol data was similar to the primary analysis (Appendix A.6). In the subgroup analysis of three trials which used both RT and chemotherapy, there was no statistically significant reduction in RID compared to placebo (RR = 0.52, 95% CI = 0.14, 1.91) (Figure 2). However, we found a statistically significant 39% reduction in RID in patients who received only RT compared to placebo (RR = 0.61, 95% CI = 0.48, 0.78) (Figure 2). 

### 3.6. Trial Sequential Analysis (TSA) for the Primary Outcome

**Figure 3:**
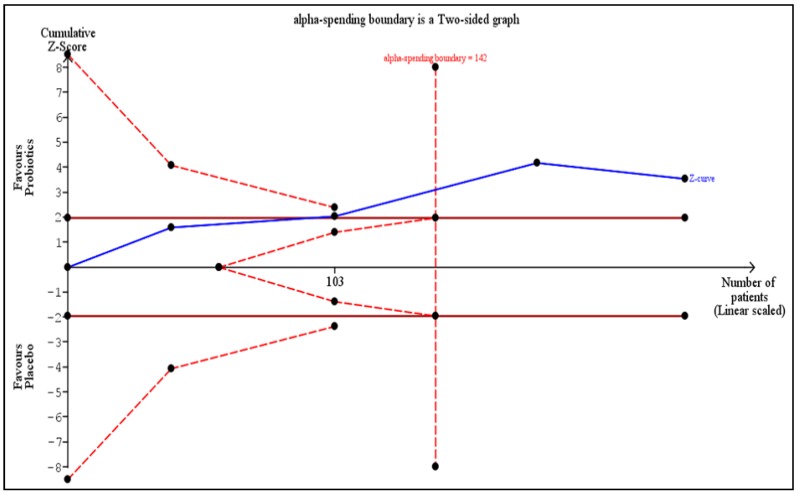
Trial sequential analysis on the incidence of radiation-induced diarrhea.

TSA on the incidence of RID is provided in Figure 3. The required heterogeneity-adjusted information size to demonstrate or reject a 38% relative risk reduction (as per Figure 1) in the incidence of RID is 717 patients (based on the meta-analysis of trials with a low risk of bias using a control event proportion of 70%, an alpha (type-1 error) of 5%, two-sided, and a beta of 20% (power = 80%)). The cumulative Z-curve (blue curve) crossed the conventional boundary (Z-statistic above 1.96) and demonstrated that probiotics significantly statistically reduced the incidence of RID as shown in our meta-analysis. Moreover, the number of patients included in our meta-analysis exceeded the required information size (that is, 142 patients), indicating that the cumulative evidence is conclusive for a 38% risk reduction in the incidence of RID.

### 3.7. GRADE Summary of Evidence

Overall, the quality of evidence based on GRADE is generally rated as low quality for the primary outcome (graded down due to possible indirectness in the dose and follow-up duration, and due to inconsistency). A more detailed description of GRADE is provided in Appendix A.7.

### 3.8. Secondary Outcomes

Compared with placebo, the use of probiotic supplementation has a significant improvement on antidiarrheal medication use (RR = 0.54, 95% CI = 0.35, 0.84, I^2^ = 57.2%) (Appendix A.8). There was no significant reduction in the incidence of soft (RR = 0.77, 95% CI = 0.48, 1.24) (Appendix A.9) and watery (RR = 0.40, 95% CI = 0.10, 1.56) (Appendix A.10) stool observed with the use of probiotic supplementation compared to placebo. 

## 4. Discussion

### 4.1. Main Findings

Our systematic review with meta-analysis has reviewed all the currently available randomized controlled trial in this important area of medicine. The use of probiotics does reduce the incidence of radiation-induced diarrhea (RID) as compared to placebo, which agrees well with previous reviews by Liu et al. and Wei et al. [5,6]. Therefore, the use of probiotics can be recommended to reduce the severity of RID in patients undergoing radiotherapy for cancers of the abdominal and pelvic cavity.

### 4.2. Comparison with Previous Meta-Analyses

Previous meta-analyses have presented low to modest benefits in the use of probiotics to reduce the incidence of radiation-induced diarrhea (RID) [5,6]. In comparison with the previous meta-analysis, which had a total of six trials of 914 participants, our study added another two trials, bringing the total number of participants to 1116 [5]. In addition, we have performed a trial sequential analysis, which confirmed the evidence.

Similarly, with another review, on grading the evidence in our study, many of the trials presented low-quality evidence [6]. This is possibly due to the fact that the trials were heterogenous and direct head-to-head comparison was not possible due to various indices such as the fact that the organisms, dose and duration were different in each of these studies.

An interesting finding in our review was that we found that for patients receiving radiotherapy with chemotherapy, probiotics are not effective in preventing RID as compared to patients receiving only radiotherapy. These findings could be explained by the fact that the incidence of RID is higher in patients receiving both chemotherapy and radiotherapy [26]. The underlying mechanism is thought to be involving gastrointestinal mucositis, which may in turn is associated with various dysfunctions such as alterations to the intestinal flora and proinflammatory cytokine production, intestinal barrier pathology and intestinal epithelial cell apoptosis [27]. A large trial investigating the various therapies for colorectal cancers both in the pre- and post-operative phase showed that the combination of chemotherapy and radiotherapy doubles the risk of grade-2 or more diarrhea from 17% to 34% [28]. Therefore, this still remains a great paradigm for doctors to ponder in patients receiving these often life-saving therapies.

### 4.3. Implications for Clinical Practice

The side effects of radiation-induced diarrhea (RID) in patients receiving radiotherapy for pelvic or abdominal cavity cancers are well established. Our study has shown that probiotics offer some hope in reducing the occurrence of these adverse events and therefore improving the patient’s quality of life.

Based on our post-hoc exploratory analysis on single or combined strains of probiotics (Appendix A.11), and with or without *Bifidobacterium* spp. (Appendix A.12), both confirmed that the probiotics do help in reducing the occurrence of RID. The use of TSA confirmed the evidence that probiotics do indeed help in reducing the occurrence of RID with the addition of two more trials as compared to the previous meta-analysis [5]. However, it is suggested that the prescription of probiotics should be made according to the patient’s needs, taking into account the current evidence, patient preferences, and cultural needs, as well as cost implications for both the patient and the government. 

### 4.4. Strengths and Limitations

The main strength of this study is that we have presented all the latest trials that look at the possible beneficial effect of probiotics in addressing the often-distressing radiation-induced diarrhea (RID). TSA has further confirmed that probiotics do indeed reduce the incidence of RID and therefore should be considered in the targeted group.

However, limitations do exist in our study. Firstly, the heterogeneity of the study may pose a problem. Looking back at all the eight trials, there were differences in the strain of probiotics used, mode of radiotherapy used, e.g., external beam or intra-cavity radiotherapy, dosage and frequency of radiations among others. Second, there are extensive differences in the study populations, including the location of study, and patient-related variables such as gender, weight, smoking status and co-morbidities, as well as the severity of the cancer and RID. For example, five studies [9,13,15,22,24] look at female patients only with gynecological cancers and three studies [12,21,23] look at abdominal cavity cancers in both genders. Another example is that Linn et al. conducted their study in a developing Asian country as compared to Salminen et al., who conducted theirs in a fully developed European country [13,22], thereby there may be significant differences in the standard of facilities and access to treatment.

Third, the criteria that weer used to determine RID differed in these studies. For example, in a few of the trials, the severity of diarrhea was determined using the National Cancer Institute Common Toxicity Criteria (NCI CTC) version 2.0 (grade 0 = none; grade 1 = an increase of <4 stools/day over pre-treatment; grade 2 = an increase of 4–6 stools/day, or nocturnal stools; grade 3 = an increase of ≥7 stools/day or incontinence or need for parenteral support for dehydration; grade 4 = physiologic consequences requiring intensive care, or hemodynamic collapse) [9,13,25], while some of the studies used the criteria set by the World Health Organization (WHO): (grade 1 = increase of 2–3 stools per day compared to pre-treatment; grade 2 = increase of 4–6 stools per day or nocturnal stools; grade 3 = increase of 7–9 stools per day or incontinence; grade 4 = increase of 10 or more stools, IV hydration needed) [12,21].

Fourth, the quality of evidence is low based on GRADE methodology, and therefore we have to interpret the results cautiously. Further, future high-quality trials are needed urgently.

## 5. Conclusions

Our study, powered with TSA, has shown that probiotics do reduce radiation-induced diarrhea (RID). Therefore, this presents as a viable option in the prevention of RID and in improving the quality of life of cancer patients receiving RID.

However, using GRADE methodology, we conclude that the quality of evidence is low. Therefore, large well-designed randomized trials with a low risk of bias are needed and the results of this study should be interpreted with caution.

## Figures and Tables

**Table 1:** The characteristics of the included studies.

First Author	Year/Area	Mean Age	Probiotics/Placebo	Probiotics with diarrhea/Placebo with diarrhea	Primary Tumor Site	Type of therapy	Total Radiation Dose	Chemotherapy
Linn	2018/Myanmar	52.5-57.38	26/28	14/23	Cervical Carcinoma	external beam pelvic radiotherapy	50Gy	Not specified
Tehrani	2016/Iran	62	22/24	7/17	Pelvic cancers (colorectal, prostate, endometrial, bladder, ovary, cervix, bone sarcoma)	conventional radiotherapy	4000 to 5000 cGy (1.8Gy/day) with 18 MV	Not specified
Salminen	1988/Finland	40–75	11/10	3/9	Cervix or uterus carcinoma	Internal and external pelvic RT and intracavitary caesium	50Gy for pelvic, 80Gy for the tumour	Intracavitary caesium
Delia	2007/Italy	No	243/239	77/124	Sigmoid, rectal or cervical cancers	Postoperative RT	60–70 Gy	Not specified
Giralt	2008/Spain	≤18	44/41	8/11	Endometrial adenocarcinoma or advanced cervical squamous cell carcinoma	Postoperative RT concomitant weekly cisplatin (only for patients with cervical cancer)	45–50.4 Gy	Weekly Cisplatin 40 mg/m^2^
Castro	2009/Brazil	NR	20/20	7/13	Cervical or endometrial cancer	RT treatments	NR	Not specified
Chitapanarux	2010/Thailand	18–65	32/31	3/14	Cervical cancer	Pelvic RT and weekly cisplatin	200 cGy per fraction, five fractions per week	Weekly cisplatin 40 mg/m^2^ for 6 weeks
Demers	2014/Canada	>18	140/86	118/80	Gynecologic, rectal, or prostate cancer	RT for gynecologic cancers without chemotherapy, gynecologic or rectal cancers with chemotherapy	40 Gy for the pelvic level	Not specified

**Table 2:** Detailed of the probiotics used.

First Author	Daily Dosage	Medication usage	Route	Timing	Probiotics Source	Diarrhea grade	Numbers of patients going into randomization	Numbers of patients assigned to treatment	Numbers of patients assigned to placebo	Genus	Species	Strain	Single or combinations	With or without Bifidobacterium
Linn	1.75 × 10^9^ CFU	t.i.d	Oral	from the first day of radiotherapy until the end of radiotherapy	Fame Pharmaceuticals, Myanmar	Common Terminology Criteria for Adverse Events	57	28	29	Lactobacillus, Bifidobacterium	L. acidophilus, B. animalis	L. acidophilus subsp LA-5, B. animalis subsp. Lactis BB-12	Combined	With Bifidobacterium
Tehrani	1.5 × 10^9^ CFU, 1.5 × 10^10^ CFU, 3.5 × 10^9^ CFU 2.5 × 10^8^ CFU, 1 × 10^10^ CFU 5 × 10^8^, CFU, 1.5 × 10^8^ CFU	b.d	Oral	one week before starting pelvic radiotherapy until the end of radiotherapy	Zist Takhmir Company, Tehran, Iran	Common Toxicity Criteria of the National Cancer Institute	52	26	26	lactobacillus, Bifidobacterium, Streptococcus	L. casei, L. acidophilus, L. rhamnosus, L bulgaricus, B. breve, B. longum, S. thermophilus,	Nil	Combined	With Bifidobacterium
Salminen	2 × 10^9^ CFU	q.d.	Oral	5 days prior to radiotherapy,10 days after finishing radiotherapy	NA	NR	24	12	12	Lactobacillus	L. acidophilus	L. acidophilus (NCDO 1748)	Single	Without bifidobacterium
Delia	1.35 × 10^12^ CFU	t.i.d.	Oral	The first day of RT until the end of therapy	VSL Pharmaceuticals, Fort Lauderdale, MD, USA	WHO grading	490	245	245	Lactobacillus & Bifidobacterium & Streptococcus	L. casei, L. plantarum, L. acidophilus, and L. delbruekii subsp. Bulgaricus & B. longum, B. breve, B. infantis, S. salivarius	S. salivrius subsp. Thermophilus	Combined	With Bifidobacterium
Giralt	3 × 10^8^ CFU	t.i.d.	Oral	One week	NR	Common Toxicity Criteria of the NCI	118	44 out of 56	41 out of 62	Lactobacillus	L. casei	Lactobacillus casei DN-114 001	Single	Without bifidobacterium
Castro	NR	NR	Oral	NR	NR	Common Toxicity Criteria of the NCI	40	20	20	Lactobacillus & Bifidobacterium	L. casei & B. breve	L. casei subsp shirota & B. breve spp.	Combined	With Bifidobacterium
Chitapanarux	4 × 10^9^ CFU	b.i.d	Oral	7 days before RT and continuing everyday during RT	Laboratio, Farmaceutico SIT, Mede, Italy	Common Toxicity Criteria of the NCI	63	32	31	Lactobacillus & Bifidobacterium	L. acidophilus & B. bifidum	L. acidophilus viv Lyophisat & B. bifidum viv Lyophisat	Combined	With Bifidobacterium
Demers	2.6 × 10^9^ CFU or 3 × 10^10^ CFU	b.i.d or t.i.d.	Oral	From the first day and ended on the last day of RT	Bifilact, Virage Santé Québec city, Canada	WHO grading	246	91	91	Lactobacillus & Bifidobacterium	L. acidophilus and B. longum	L. acidophilus LAC-361 and B. longum BB-536	Combined	With Bifidobacterium

Note, t.i.d (three times a day); b.i.d (two times a day); q.d (everyday); CFU (colony-forming unit); Percentage a (percentage of patients who completed the treatment; Percentage b (percentage of patients who completed the placebo).

**Figure A1:**
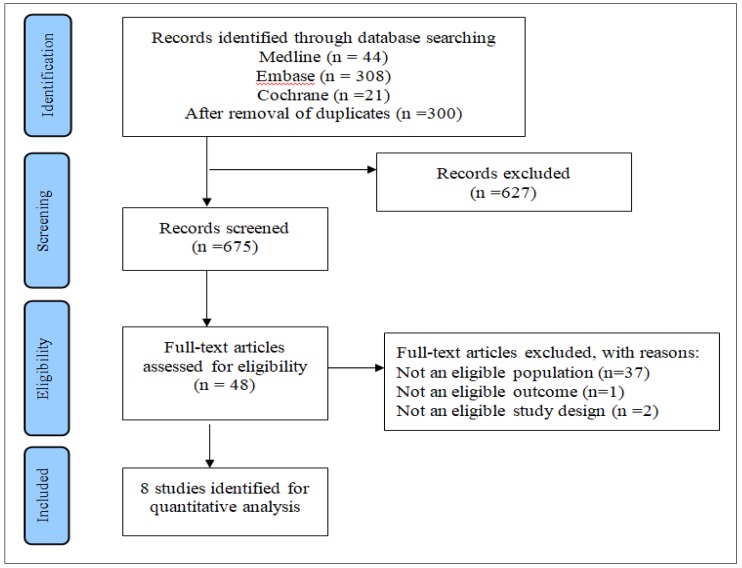
Preferred Reporting Items for Systematic Reviews and Meta-Analyses (PRISMA) flow diagram of the literature screening process.

## Abbreviations

GRADE	Grading of Recommendations, Assessment, Development and Evaluation
RCT	Randomized controlled trials
RID	Radiation-induced diarrhea
RT	Radiation therapy
TSA	Trial sequential analysis

## Appendix A

### Appendix A.1. Search Strategy in MEDLINE

		**Medline**	**Embase**	**Cochrane**
1	exp DIARRHEA/	49,612	228,556	3029
2	exp RADIOTHERAPY/	168,386	479,346	5602
3	Radiation-induced Diarrhea.mp.	53	72	30
4	exp PROBIOTICS/	13,609	28,098	1590
5	exp SYNBIOTICS/	356	1200	96
6	exp LACTOBACILLUS/	25,693	39,707	1365
7	exp BIFIDOBACTERIUM/	5163	10,399	552
8	exp SACCHAROMYCES/	104,336	98,961	138
9	exp Enterococcus/	18,010	43,801	249
10	exp BIFIDOBACTERIUM/	5163	10,399	552
11	exp Randomized Controlled Trials as Topic/	119,100	146,594	7449
12	exp Clinical Trial/	800,652	1,329,589	173
13	random$.ab.	956,882	1,275,640	599,733
14	4 or 5 or 6 or 7 or 8 or 9 or 10	156,392	196,258	2516
15	1 or 2 or 3	217,601	697,441	8569
16	11 or 12 or 13	1,460,326	2,207,279	601,980
17	14 and 15 and 16	485	2382	229
18	limit 17 to (humans and yr = “2016–2018”)	44	308	21

### Appendix A.2. PRISMA Flow Diagram

#### Description of Included Trials

Study selection, inclusion, and exclusion at each screening phase for the efficacy end points are described in Figure A1 (PRISMA flowchart).

### Appendix A.3. Assessment of Primary Outcome (The Funnel Plot Asymmetry Test)

Inference: evidence of publication bias due to asymmetry in the funnel plot. Due to the limited number of studies (<10 studies) included, interpret these results with caution.

### Appendix A.4. Egger’s Regression Test

Since *p*-value is more than 0.1, this indicates weak evidence of small-study effects.

### Appendix A.7. GRADE Summary of Evidence

**Table A1:** GRADE Summary of Evidence.

Certainty Assessment							№ of Patients		Effect		Certainty	Importance
№ of Studies	Study Design	Risk of Bias	Inconsistency	Indirectness	Imprecision	Other Considerations	Probiotics	Placebo	Relative (95% CI)	Absolute (95% CI)		
Outcome: Incidence of Radiation-Induced Diarrhea												
Three	Randomized trials	Not serious ^a^	Serious ^b^	Serious ^c^	Not serious ^d^	None ^e^	107/215 (49.8%)	105/151 (69.5%)	RR 0.61 (0.39 to 0.96)	271 fewer per 1000 (from 424 fewer to 28 fewer)	⨁⨁◯◯ LOW	IMPORTANT

a: Low risk of bias trials were used for GRADE; b: presence of heterogeneity; c: presence of indirectness due to difference in probiotics, its dose, duration of follow-up, etc. (refer characteristics table); d: required information size is reached as per TSA; e: no small-study effects as per Egger’s regression test.
